# Transcriptome analysis discloses antioxidant detoxification mechanism of *Gracilaria bailinae* under different cadmium concentrations and stress durations

**DOI:** 10.3389/fpls.2024.1371818

**Published:** 2024-07-05

**Authors:** Zailiang Li, Yangmei Li, Enyi Xie, Yuchun Shen

**Affiliations:** Laboratory of Zhanjiang Key Marine Ecology and Aquaculture Environment, Fishery College, Guangdong Ocean University, Zhanjiang, China

**Keywords:** *G. bailinae*, cadmium stress, antioxidant, gene expression, antioxidant detoxification mechanism

## Abstract

To remedy Cd pollution in the ocean, macroalgae are used as a bioremediation tool because of their ability to absorb and accumulate Cd. *Gracilaria bailinae* has high economic and ecological value and can survive in Cd contaminated waters; however, the underlying molecular mechanisms remain unclear. In this study, physiological and biochemical indexes were analyzed after 1, 3, 5, or 7 days of Cd^2+^ exposure; further, the transcriptome of *G. bailinae* was examined after a 7-day exposure to a Cd^2+^ culture environment with Cd levels of 0 mg L^-1^ (cd1, control), 1 mg L^-1^ (cd2, low concentration), and 2.5 mg L^-1^ (cd3, high concentration). The results showed that in the cd2 group, *G. bailinae* maintained a stable RGR that did not differ significantly *(P* > 0.05) from that of the cd1 group. However, the soluble protein and MDA contents, as well as the activities of SOD, CAT and POD, were significantly increased (*P*< 0.05) compared to the cd1 group. No significant differences (*P* > 0.05) were found among the different Cd^2+^ stress durations. In contrast, compared with the cd1 group, the RGR, soluble protein content, SOD, CAT, and POD activities were significantly decreased (P< 0.05), while the MDA content was significantly increased (P< 0.05) in the cd3 group. Furthermore, significant differences (*P*< 0.05) were observed among the various tested Cd^2+^ stress durations within the cd3 group. Compared to the cd1 group, a total of 30,072 DEGs and 21,680 were identified in the cd2 and cd3 treatments, respectively. More up-regulated genes were found in cd2 group than in cd3 group. GO enrichment analysis showed that these genes were related to peptidase activity, endopeptidase activity, ion transport, peptide biosynthetic and metabolism. In addition, DEGs related to histidine metabolism and the stilbene, diarylheptane, and gingerol pathways were significantly up-regulated in the cd2 group compared to the cd3 group, which resulted in enhanced activities of antioxidant enzymes and promoted cell wall regeneration. The results of this study reveal the response mechanism of *G. bailinae* to Cd^2+^ stress, providing valuable insights for assessing the bioremediation potential of *G. bailinae* for Cd-contaminated waters.

## Introduction

Cadmium(Cd) is a toxic heavy metal commonly found in industrial effluents, mining wastewater and agricultural runoff. Due to its high toxicity and ubiquity in various industrial processes, Cd is considered a model pollutant (Ma et al., 2018; Hamid et al., 2023). In offshore waters, Cd is one of the factors leading to heavy metal pollution (Cai et al., 2012). Because of its characteristics of long residue time, difficult degradation, irreversible harm, easily causes serious damage to the marine ecological environment, and even poses adverse effects on human health through accumulation across the food chain (Wu et al., 2007; Duan et al., 2021), Cd pollution in coastal waters has attracted scholarly attention both in China and internationally, and has rapidly become a hot topic in the fields of marine ecology, environmental science, and ecotoxicology (Luparello et al., 2012).

Previous studies have shown that macroalgae have a relatively strong ability to absorb and accumulate Cd and can thus be used as an effective bioremediation tool of marine Cd pollution (Deng et al., 2015). The absorption and accumulation of heavy metals by macroalgae are mainly achieved through the mutual adsorption of functional groups on the cell wall of algae and heavy metal ions. The polysaccharides of the cell wall of algae contain some functional groups, such as hydroxyl, carboxyl, amino, sulfhydryl and phosphoric acid, which can be combined with heavy metal ions in water (Parker et al., 1998; Hamid et al., 2023). Chitosan (CT) is a natural polysaccharide that is easily extracted from chitin in plant cell walls and is the second most common polymer after cellulose (Abd Malek et al., 2021; Hamid et al., 2023). It is a biocompatible, non-toxic and renewable material with free CT hydroxyl and amino groups, and its high abundance, low cost, biocompatibility, biodegradability, non-toxicity and adsorption capacity make it a viable heavy metal removal adsorbent (Zhou et al., 2013; Zhang et al., 2019; Hamid et al., 2023).

However, Cd—a non-essential element for plant growth and development—has been reported to be highly toxic for different organisms even at extremely low concentrations, including both marine and freshwater algae (Nriagu and Pacyna, 1988; Suárez et al., 2010). Cd stress can induce toxic oxidative stress in algae, which can increase the content of reactive oxygen species (ROS) in the algal body (Liu et al., 2011). Relevant ROS include O_2_·^-^, H_2_O_2_, and OH·, which can result in an imbalance between oxidative and antioxidant systems. When the degree of oxidative stress exceeds the scavenging capacity of algae cells for ROS, tissue damage can occur, resulting in membrane lipid peroxidation, damage to the membrane system, and an increase in malondialdehyde (MDA) content (Lushchak, 2011; Huang, 2013). MDA is the end product of membrane lipid peroxidation caused by ROS accumulation, and its content can reflect the degree of adversity injury to plants (Weismann et al., 2011; Wang et al., 2014). Moreover, algae that are subject to Cd stress in a certain concentration range can increase soluble protein content and enhance the activities antioxidant enzymes such as superoxide dismutase (SOD), catalase (CAT), and peroxidase (POD), which can reduce the content of ROS, control the content of MDA, and relieve the oxidative toxic effects caused by Cd exposure (Lü et al., 2017). SOD is the first antioxidant enzyme to engage in the ROS scavenging reaction, which can rapidly dismutate O_2_·^-^ into H_2_O_2_ and O_2_ (Miura et al., 2012; Mccord and Fridovich, 1969). CAT is essential for scavenging H_2_O_2_, which is produced when cells are under stress, and it has the ability to directly catalyze the disproportionation reaction of H_2_O_2_, degrading it into H_2_O and O_2_ (Leung, 2018). POD primarily catalyzes the efficient transfer of H_2_O_2_ and organic peroxides from the cytoplasm to recipient molecules. This process effectively prevents the harmful accumulation of hydrogen peroxide and organic peroxides within cells, thereby safeguarding cellular health (Zamocky and Dunand, 2006; Hua et al., 2018). However, when the concentration of cadmium exceeds the tolerance threshold of macroalgae, it exerts toxic effects on the algae, disrupts the metabolism of algal cells, and even affects the regulation of gene expression. This leads to stagnation of growth, decreased synthesis of bioactive substances, and an increase in the activity of antioxidant enzymes such as SOD, CAT, and POD (Zhu, 2010; Huang, 2013).

*Gracilaria bailinae* is a macroeconomically important red algae, which can be found in abundance in the coastal waters of Guangdong and Hainan provinces in China (Xu et al., 2021; Huang et al., 2023). *G. bailinae* has broad application prospects in mariculture and marine ecological restoration (Xu et al., 2021; Huang et al., 2023). At a Cd concentration of 5 mg.L^-1^ and a stress duration of 7 days, the growth, internal cellular structure, photosynthesis, pigment content, antioxidant enzyme activity, and lipid peroxidation level of *G.bailiniae* are significantly increased (Huang et al., 2022). However, in oceanic areas polluted by Cd, the oxidative stress Cd imposes on algae is continuous and dynamic, and the antioxidant detoxification mechanism of algae in response to Cd stress is influenced by both Cd concentration and the duration of stress exposure (Yu et al., 2007), and no detailed and comprehensive report on the antioxidant response and related gene expression of algae under different concentrations and stress durations of Cd has been published to date. Further, the molecular mechanisms underlying the reaction of *G. bailinae* to Cd stress remain unknown. The present study specifically aims to disclose the antioxidant physiological and biochemical response mechanism and related gene expression at the transcriptional level by using the RNA-seq technique of *G. bailinae* under different concentrations and stress durations of Cd. In the context of marine heavy metal pollution, the results of this paper may provide theoretical guidance for academic research and practical applications related to the bioremediation of marine heavy metal pollution with macroalgae.

## Materials and methods

### Material source and pretreatment

In November 2022, wild *G. bailinae* were collected from the Wushi coastal sea area (N 20°33′, E 109°51′), which is affiliated with Zhanjiang of Guangdong Province, China. Samples were transported back to the laboratory within 2 h at a low temperature of about 4°C. In the laboratory, algal surface attachments were removed and samples were temporarily cultured in a 20 L tank filled with natural seawater (disinfected and filtered), and kept aerated for 24 h. The temporary culture temperature was 23°C, the photoperiod was 12:12-h light: dark cycle, the light intensity was 4000 Lux, and the pH value was 8.1. The medium was changed every 2 days. Healthy algal bodies with strong growth and consistent size were selected before the start of the experiment. The same part of these algal samples (middle and upper torso) was cut into small sections of the same size for the experiment.

### Experimental method

To ensure stable experimental conditions, the experiments were carried out in an incubator under constant temperature and lighting. The culture conditions, such as the experimental temperature, light, cultured seawater, and pH value, were equal to those of the temporary culture conditions. A total of 2.5 ± 0.003 g of fresh algae was weighed for experimental culture, and the culture container was a 1.2 L triangle flask filled with 1 L of cultured seawater. The triangle flask was capped with tin foil and kept aerated for 24 h. To ensure adequate and suitable nutrient conditions, the concentrations of nitrogen and phosphorus in the cultured seawater were adjusted to 100 and 10 μmol·L^-1^, respectively. The medium was changed every second day, and the total duration of the experiment was 7 d. According to the needs of the experiment, the fresh weight of the algal body was measured on the 1st, 3rd, and 5th days. A certain number of algal branches were trimmed to maintain the initial weight (2.5 ± 0.003 g) and then returned to continue the culture.

### Group design and sampling

with a large number of our preliminary experiments, we found that *G. bailinae* showed relatively strong heavy metal tolerance especially for Cd concentrations of less than or equal to 2.5 mg·L^-1^, and its relative growth rate was higher in the treatment group with Cd^2+^ concentration of 1 mg.L^-1^ than in the treatment group with Cd concentration of 2.5 mg.L^-1^. Therefore, in this experiment, three treatment groups with Cd^2+^ (CdCl_2_) concentrations of 0, 1, and 2.5 mg·L^-1^ with three biological replicates were set up. Each treatment group used 18 parallels, three of which were fixed to monitor the relative growth rate (RGR) of algal bodies. The fresh weight of algal bodies was measured at the end of the 1st, 3rd, 5th, and 7th days. Twelve parallels were used to measure physiological and biochemical indexes, and samples were taken at the end of the 1st, 3rd, 5th, and 7th days. The remaining three parallels were used for transcriptomic analysis, and the samples were taken after 7 d of culture. For convenient data processing, the treatment groups 0, 1, and 2.5 mg·L^-1^ were numbered and marked as cd1, cd2, and cd3, respectively, of which the 0 mg·L^-1^ treatment group was designated as the control group.

### Growth measurements

Following the method of Yong et al. (2013), the RGR of *G. bailinae* was calculated according to the following formula:


$$\text{RGR }\left(\%{\text{d}}^{-1}\right)=\left(\text{ln Mn}-\text{ln M}0\right)/\text{t}\times 100\%$$

where M0 represents the initial weight of *G. bailinae* and Mn represents its weight after the n-th day of experimental culture.

### The soluble protein, MDA contents and the activities of SOD, CAT, POD measurements

At the end of the 1st, 3rd, 5th, and 7th days, 0.1 g of algal tissue was taken, and 1 mL of extract solution was added into an ice bath homogenate. This mixture was centrifuged at 12000 rpm for 10 min at 4°C. The soluble protein, MDA contents, and the activities of SOD, CAT, POD were measured using the commercial assay kits (Grace, Suzhou, China).

The soluble protein measurement principle is that in an acidic solution, Coomassie Blue G-250 combines with protein to form a blue complex. Through spectral scanning, the blue complex has a maximum absorption peak at 600nm, and its color depth is proportional to the protein content within a certain protein concentration range (1-1000μg.mL^-1^). The calculation of the standard curve for soluble protein is as follows:


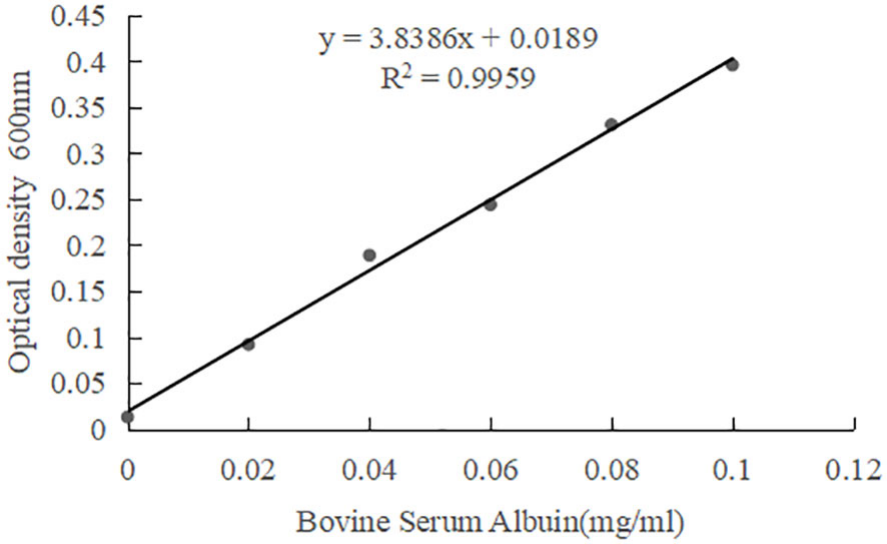


x is the concentration of protein standard (mg/ml); y is the difference in absorbance values between the test group and the blank group at 600 nm.

The MDA content measurement principle is that MDA condenses with thiobarbituric acid under high temperature and acid conditions, producing red products with a maximum absorption peak at 532nm. The peroxidation in the sample can be estimated after colorimetry of the lipid content. At the same time, the absorbance at 600nm is measured, and the MDA content is calculated by the difference between the absorbance at 532nm and 600nm. The MDA content is calculated according to the following formula:


MDA content (nmol/gFW)=[ΔA÷(ε×d)×V2×109]÷(W×V1÷V)

V represents the total volume of the sample extract, which is 1 mL; V1 represents the volume of the sample added to the reaction system, which is 0.2 mL; V2 represents the total reaction volume of the sample extract and working solution, which is 5×10^-4^ L; d is the optical path length of the cuvette, 0.5cm; ϵ is the molar extinction coefficient of MDA, 155×10^3^ L/mol/cm; W represents the mass of the sample, in grams (g).

The SOD activity measurement principle is that WST-8 can react with the superoxide anion (O_2_.-) catalyzed by Xanthine Oxidase to produce a water-soluble methyl dye, which has a maximum absorption at 450nm. SOD can rapidly dismutate O_2_·- into H_2_O_2_ and O_2_, thus restraining the formation of formazan. The deeper the color of the reaction solution, the lower the SOD activity, and vice versa, the higher the activity level.

SOD enzyme activity unit(U) definition: In the xanthine oxidase coupled reaction system, when the inhibition rate is 50%, the SOD enzyme activity in the reaction system is defined as one enzyme activity unit.

The CAT activity measurement principle is that CAT catalyzes hydrogen peroxide to produce water and oxygen, and the remaining hydrogen peroxide is color-developed with a new color development probe, which has a maximum absorption peak at 510nm. The CAT enzyme activity in the sample was calculated by reducing the amount of H_2_O_2_.The calculation of the standard curve for H_2_O_2_ is as follows:


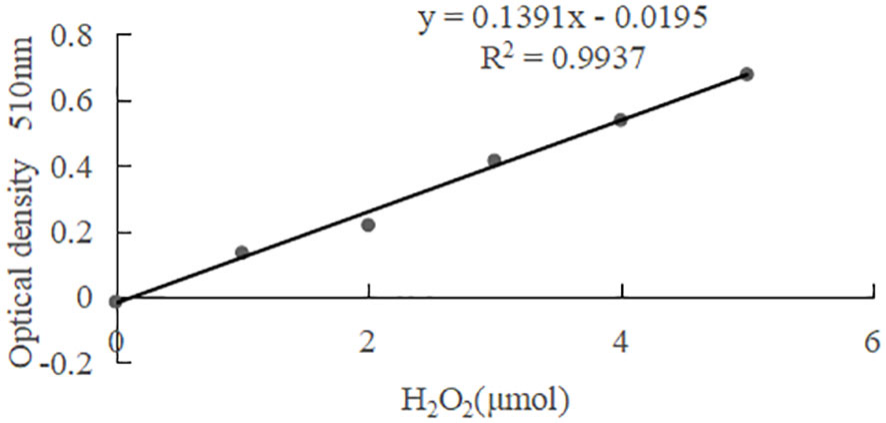


x is the concentration of H_2_O_2_ standard (μmol); y is the difference in absorbance values between the test group and the blank group at 600 nm.

CAT activity Unit(μmoL/min/g FW) definition: CAT and decomposing 1 μmol of H_2_O_2_ per gram of tissue per minute is defined as one unit of enzyme activity.

The POD activity measurement principle is that under the catalysis of peroxidase, H_2_O_2_ oxidizes guaiacol to produce a reddish-brown product, which has the maximum light absorption at 470nm. Therefore, peroxidase activity can be determined by measuring the change in the light absorption value at 470 nm.

POD Enzyme Activity Unit (△OD470/min/g FW) definition: An increase in absorbance of 1 at 470 nm in the reaction system per minute per gram of tissue is defined as one unit of enzyme activity.

All measurements were made using a microplate reader, and the experimental procedure and calculation formula were followed according to the instructions provided with the manufacturer’s kit.

### Total RNA extraction, cDNA library construction, and transcriptome sequencing

After 7 d of treatments cd1, cd2, and cd3, the Trizol kit (Invitrogen, Carlsbad, CA, USA) was used to isolate the total RNA of *G. bailinae*. The total RNA was validated via RNase-free agarose gel electrophoresis and the quality of RNA was analyzed by Agilent 2100 Bioanalyzer (Agilent Technologies, Palo Alto, CA, USA). Then, the mRNA of *G. bailinae* was enriched with Oligo (dT) beads and cleaved into small fragments by fragment buffer. These fragments were then reverse-transcribed into cDNA. Subsequently, a second strand of cDNA was generated using DNA polymerase I, RNase H, dNTP, and buffer. After purification with the Qiagen rapid PCR extraction kit (Qiagen, Venlo, Netherlands), cDNA fragments were ligated to the Illumina sequencing adapter following end repair and poly(A) addition. Ligation products were size selected via agarose gel electrophoresis, amplified via PCR, and sequenced using Illumina NovaSeq 6000 (Gene Denovo Biotechnology Co., Guangzhou, China).

### Analysis of the relationship between samples

Principal component analysis of all nine transcriptome datasets was performed using R (http://www.r-project.org/) to examine differences in expression patterns between different samples. Three replicates were performed for each Cd^2+^ concentration treatment, and the reproducibility of transcriptome data was assessed by Pearson correlation analysis.

### Filtering of clean reads, *de novo* assembly, and annotation

The original reading was pruned using fastp (version 0.18.0) to remove bases containing adapters, more than 10% of unknown nucleotides, and more than 50% of low-quality bases (q value ≤ 20) (Chen et al., 2018). *De novo* assembly of the transcriptome was performed using the short-read assembly program Trinity (Grabherr et al., 2011). Using an e-value threshold of 105, BLASTx (http://www.ncbi.nlm.nih.gov/BLAST/) was used to compare transcripts to the NCBI non-redundant (Nr) transcript and protein database (http://www.ncbi.nlm.nih.gov), the Swiss-Prot protein database (http://www.expasy.ch/sprot), the Kyoto encyclopedia of genes and genomes database (KEGG) (http://www.genome.jp/kegg), and the COG/KOG database (http://www.ncbi.nlm.nih.gov/COG). Unigenes were annotated. Then, protein function annotations were obtained according to the best comparison results.

### Differential gene expression analysis

DESeq2 (Love et al., 2014) and edgeR (Robinson et al., 2010) were used to analyze the differential expressions of RNA between different groups and samples, respectively. Genes with a false discovery rate of less than 0.001 and an absolute multiple change of ≥ 4 are considered differentially expressed genes (DEGs).

### Verification via real-time quantitative PCR

Eleven genes associated with Cd^2+^ concentration changes were randomly selected from the transcriptome results. The 18s RNA gene (Liu et al., 2021; Huang et al., 2023) was used as internal reference gene to verify the transcriptome. The reaction steps are as follows: 95°C reaction for 300 s, 95°C reaction for 10 s, 60°C reaction for 15 s, and 72°C reaction for 15 s; then, the fluorescence signal was read, followed by 95°C reaction for 10 s, 65°C reaction for 60 s, and 95°C reaction for 1 s. Each reaction was performed three times. Primers were designed by Primer-BLAST using the website of the national center for biotechnology information (https://www.ncbi.nlm.nih.gov/tools/primer-blast/, December 2023). Relative transcription levels were measured by the 2^-△△^Ct method (Qin et al., 2021; Huang et al., 2023). Primer sequences and qRT-PCR validation results are shown in **Supplementary Table S1**.

### Statistical analyses

The data of RGR, soluble protein and MDA content, SOD, CAT and POD activities were expressed by means ± SD (n ≥ 3). SPSS 26 (IBM Corp., Armonk, NY, USA) was used to run one-way ANOVA and Duncan’s tests to evaluate the statistical significance of different treatments. The significance level was *P*< 0.05.

## Results

### RGR and soluble protein content of *G. bailinae* under different concentrations and stress duration of Cd^2+^

**Figure 1:**
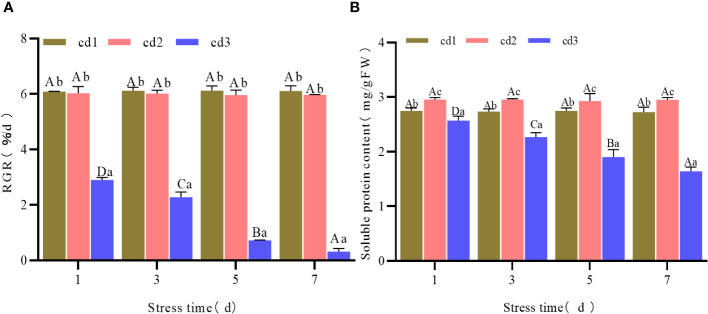
RGR and Sp content of *G. bailinae* under different concentrations and stress duration of Cd^2+^. cd1 **(A)** RGR. **(B)** soluble protein content. Different capital letters indicate that the same treatment group was significantly different (*P*<0.05) among different stress duration, and different lowercase letters indicate that different treatment groups was significantly different (*P*<0.05) in the same stress duration. cd1 represents the control group, cd2 represents the treatment group with a Cd^2+^ concentration of 1 mg.L^-1^, cd3 represents the treatment group with a Cd^2+^ concentration of 2.5 mg.L^-1^(The same hereinafter).

Under the same stress duration but different Cd^2+^ concentrations, RGR decreased with increasing Cd^2+^ concentration. No significant difference was found between the cd2 and cd1 treatment groups (*P* > 0.05), but a significant difference (*P*< 0.05) was found between the cd3 and cd1 treatment groups (**Figure 1A**). The soluble protein of *G. bailinae* first increased and then decreased with increasing Cd^2+^ concentration, and was significantly different (*P*< 0.05) among Cd^2+^ treatment groups (**Figure 1B**). In the control group cd1 and the treatment group cd2, the RGR and soluble protein contents of *G. bailinae* remained relatively stable with increasing Cd^2+^ stress duration. No significant difference (*P* > 0.05) was found among different stress durations (**Figures 1A, B**). In the treatment group cd3, the RGR and soluble protein contents of *G. bailinae* decreased with increasing stress duration, and significant differences (*P*< 0.05) were found among different Cd^2+^ stress durations (**Figures 1A, B**).

### MDA contents and SOD, CAT, POD activities of *G. bailinae* under different concentrations and stress duration of Cd^2+^

**Figure 2:**
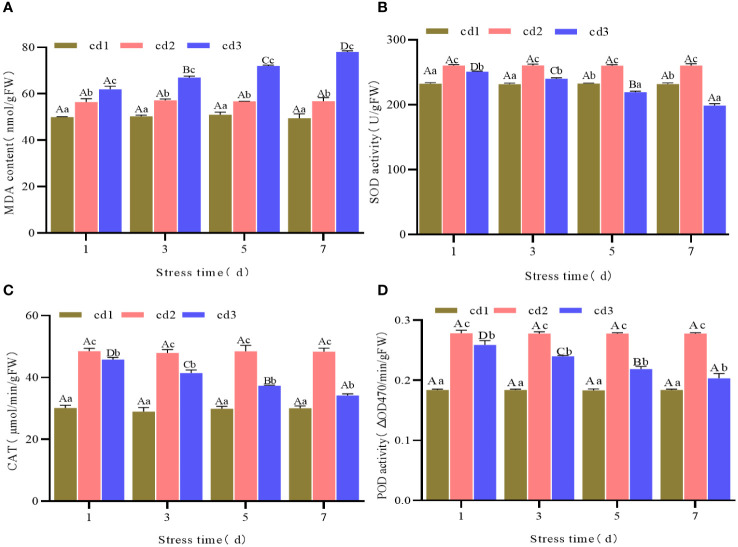
MDA content and SOD, CAT, POD activities of *G. bailinae* under different concentrations and stress duration of Cd^2+^. **(A)** MDA content. **(B)** SOD activity. **(C)** CAT activity. **(D)** POD activity. Different capital letters indicate that the same treatment group was significantly different(P <0.05) among different stress duration, and different lowercase letters indicate that different treatment groups was significantly different (*P* <0.05) in the same stress duration.

Under the same stress duration but different Cd^2+^ concentrations, the content of MDA of *G. bailinae* increased with increasing Cd^2+^ concentration. Differences were significant (*P*< 0.05) among different Cd^2+^ concentration treatment groups. In the control group cd1 and the treatment group cd2, MDA contents were relatively stable with extended stress duration and no significant differences (*P* > 0.05) were found among different stress durations (**Figure 2A**). In the treatment group cd3, the MDA content increased with extended stress duration, and significant differences were found among different stress durations(*P*< 0.05) (**Figure 2A**). The activities of SOD, CAT, and POD increased first and then decreased with increasing Cd^2+^ concentration under the same stress duration. Significant differences (*P*< 0.05) were found among different Cd^2+^ concentration treatment groups (**Figures 2B–D**). In the control group cd1 and the treatment group cd2, the activities of SOD, CAT, and POD were relatively stable over extended stress duration. No significant differences (*P* > 0.05) were found among different stress durations (**Figures 2B–D**). In the treatment group cd3, the activities of SOD, CAT, and POD decreased with extended stress duration, and significant differences (*P<* 0.05) were found among different stress durations (**Figures 2B–D**).

### Data quality summary

Clean data were obtained after quality control of the raw data of nine samples were obtained by sequencing (**Supplementary Table S2**). A total of 406,561,034 raw data were obtained, and 403,855,016 clean data were obtained after removing joints and low-quality sequences. The clean data content of different samples exceeded 99%, the GC content ranged between 50.24% and 51.56%, Q20 exceeded 97%, and Q30 exceeded 93%. These results indicate that the overall quality of transcriptome sequencing data from *G. bailinae* was good and met the needs of follow-up analyses. The clustering of the assembled transcripts of *G. bailinae* yielded a total of 58,955 single genes (>200 bp), N50 was 15,093, and the length was 2,260 base pairs. Individual genes ranged in size from 202 to 30,299 bp, with an average length of 1,965 bp and a total length of 115,859,584 (**Supplementary Table S3**).

### Evaluation of data reproducibility

Pearson correlation coefficient (r) among three repeated samples for each treatment was ≥0.998 (r = 0.8 indicates strong correlation) (**Supplementary Figure S1A**). The results of principal component analysis showed that the expression patterns of *Betula alba* differed in different Cd^2+^ treated groups (**Supplementary Figure S1B**). In addition, qRT-PCR and mRNA-Seq analyses showed that 10 randomly selected genes had similar expression tendencies (**Supplementary Table S1**).

### Gene functional annotation

At present, the whole genome sequencing of *G. bailinae* has not been completed (Huang et al., 2023). To obtain transcriptome information of *G. bailinae*, the BLASTx comparison tool was used to compare the assembled 58,955 gene sequences with the NCBI non-redundant protein databases Nr, Swiss-Prot, KEGG, and COG/KOG (e value< 0.00001). The protein with the highest sequence similarity to Unigene was identified, from which the Unigene functional annotation information was obtained. Among the 58,955 gene sequences obtained after transcriptome assembly, 42,943 genes could be compared with these four major databases: 41,966 genes were compared with the Nr database, 29,708 genes were compared with the Swiss-Prot database, and 31,038 genes were compared with the KEGG database. The number of genes compared with the COG/KOG database was 26,105 (**Supplementary Figure S2**).

In the Nr database, the main matches were *Nitzschia inconspicua* and *Gracilariopsis chorda*, which accounted for 32.56% and 20.29%, respectively. This result indicates that the homology of the *G. bailinae* unigene (obtained under Cd^2+^ stress) was low compared with that of known *Gracilaria* species in public databases; it was necessary to enrich the gene data resources of *G. bailinae* (**Supplementary Figure S3**).

Metabolism, genetic information processing, environmental information processing, cellular processing, and organismal systems were the five primary functional categories of genes that matched the KEGG database. Each category was subdivided according to secondary groups of functions. In particular, the largest number of unigenes were involved in “global and overview maps” (6,412), followed by “translation” (3,968), “carbohydrate metabolism” (2,430), and “folding, sorting, and degradation” (2,250) (**Supplementary Figure S4**).

Genes that matched the KOG database were divided into 25 groups. The largest group was “posttranslational modification, protein turnover, chaperones” (4,713), followed by “translation, ribosomal structure and biogenesis” (4,519), “general function prediction only” (3,909), “signal transduction mechanisms” (3,593), and “energy production and conversion” (2,156) (**Supplementary Figure S5**).

Based on the Nr annotation information, Blast2GO was used to obtain GO function annotations (Ashburner et al., 2000; Conesa et al., 2005; Huang et al., 2023). Three ontologies in GO were used to categorize the molecular functions, cellular component, and biological process of genes. The unigenes that were annotated into the GO database by GO annotation were classified into 50 subclasses. The top three subclasses of biological process were cellular process, metabolic process, and localization, with 24,990, 22,130, and 6,341 genes, respectively. The top three subclasses of cellular component were cellular anatomical entity, protein-containing complex, and virion component, with 15,697, 7,325, and 166, respectively. The top three subclasses of molecular functions were catalytic activity, binding, and transporter activity, with 20,111, 19,572, and 3,533 genes, respectively (**Supplementary Figure S6**).

### Analysis of DEGs

#### Overview of DEGs under stress

**Figure 3:**
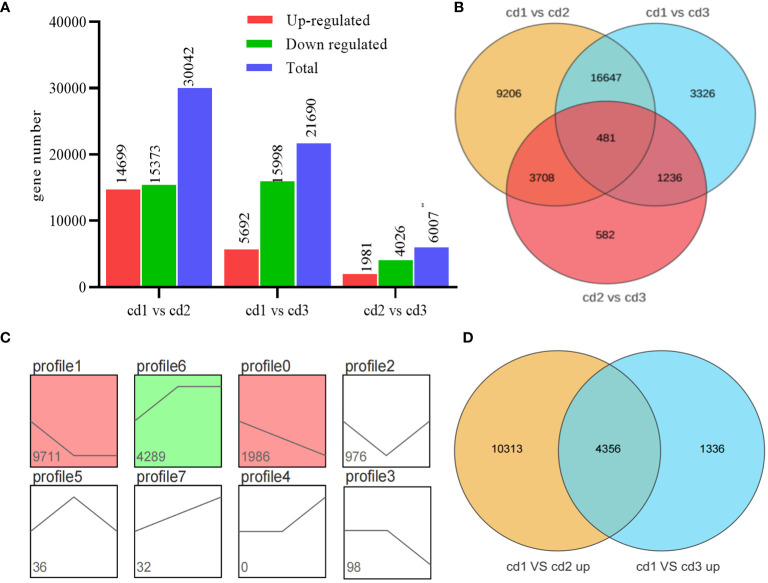
Comparative transcriptome analysis of *G. bailinae* under different Cd^2+^ treatments. **(A)** Gene number analysis of DEGs in cd1 VS cd2, cd1 VS cd3 and cd2 VS cd3 comparisons. **(B)** Venn diagram depicting the gene expression in cd1 VS cd2, cd1 vs cd3 and cd2 VS cd3 comparisons. **(C)** Trend analysis of DEGs in cd1 VS cd2 and cd1 VS cd3. Significant enrichment trends are shown by colored blocks (P< 0.05). The bottom-left number represents the number of genes. **(D)** Venn diagram depicting the up-regulated gene number in the cd1 VS cd2 and cd1 VS cd2 comparisons.

Using log2 (FC) ≥ 2 and false discovery rate ≤ 0.001 as screening conditions, DEGs were identified between Cd^2+^ treatment groups and the control group (cd1 VS cd2; cd1 VS cd3), as well as between the two Cd^2+^ treatment groups (cd2 VS cd3, **Figure 3A**). In total, 14,669 and 15,373 genes exhibited different levels of expression between cd1 and cd2, while 5,692 and 15,998 DEGs were found between cd1 VS cd3. Among these DEGs, 17,128 (16,647 + 481) were shared between the two Cd^2+^ treatment comparisons with the control group. A total of 481 of these were sensitive to Cd^2+^ concentration as they were also differentially expressed between the cd2 and cd3 treatment groups (**Figure 3B**). To further explore the gene expression patterns of *G. bailinae* treated with different Cd^2+^ concentrations, the gene expression trends of 17,128 DEGs were analyzed. Significant differences (*P*< 0.05) are summarized in the colored graph. All DEGs were clustered into eight trend profiles, where profile1, profile6, and profile0 have significant differences, among which profile1 and profile0 form a large part of DEGs (11,697 and 17,182, respectively). About 68.1% of DEG expressions decreased with increasing Cd^2+^ concentration (**Figure 3C**). **Figure 1A** shows that more down-regulated genes (15,373 and 15,998) were found than up-regulated genes (14,669 and 5,692) in algae of the Cd^2+^ treatment group, especially in cd1 VS cd3; however, more up-regulated genes were found in cd2 (14,669) than in cd3 (5,692). Among these up-regulated genes, 4,356 were shared between the two Cd^2+^ treatment comparisons with the control, 10,313 were especially expressed in cd2, and 1,336 were especially expressed in cd3 (**Figure 3D**).

### GO enrichment and KEGG enrichment

**Figure 4:**
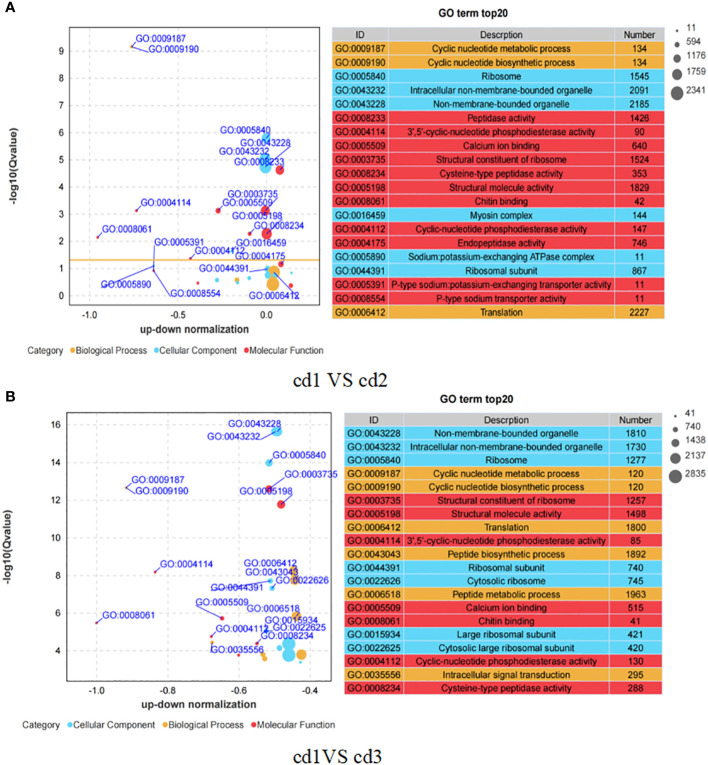
Differential GO enrichment pathway analysis of *G. bailinae* in cd1 VS cd2 and cd1 VS cd3. **(A)** GO enrichment pathway analysis in cd1 VS cd2. **(B)** GO enrichment pathway analysis in cd1 VS cd3.

GO enrichment analysis was conducted to identify the major functional categories of different genes using cluster Profiler software (Yu et al., 2012). **Figure 4** shows that the top 20 enriched GO terms in cd1 VS cd2 and cd1 VS cd3 contained three fundamental GO categories, namely, biological process, cellular process, and molecular process. Of these GO terms, more (11 in 20) GO entries involved in molecular function were enriched in cd1 VS cd2 (**Figure 4A**). These entries were mostly related to peptidase activity, endopeptidase activity, and ion transport. In the cd1 VS cd3 group, more GO items were enriched in biological processes, most of which were related to peptide biosynthetic and metabolism, as well as intracellular signal transduction (**Figure 4B**).

**Figure 5:**
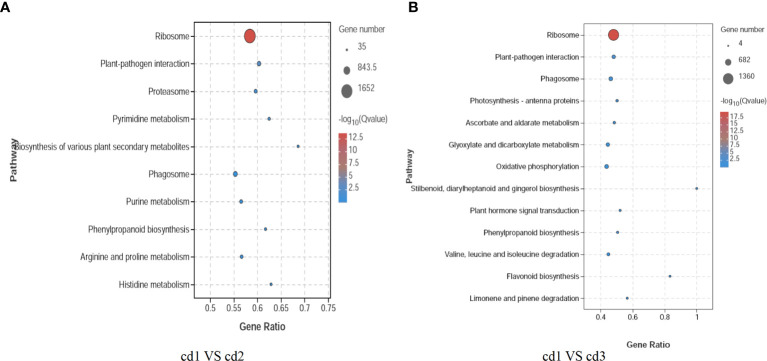
Differential KEGG enrichment pathway analysis of *G. bailinae.* **(A)** KEGG enrichment pathway analysis in cd1 VS cd2. **(B)** KEGG enrichment pathway analysis cd1 VS cd3.

In addition, to understand the biological functions and signal transduction pathways of these DEGs, the KEGG pathway database was used to identify DEG-related pathways under Cd stress. As shown in **Figure 5**, in the comparisons of cd1 VS cd2 and cd1 VS cd3, there are 10 and 13 significantly enriched pathways, respectively. Four enriched pathways are shared between cd1 VS cd2 and cd1 VS cd3, which are Ribosome, Plant-pathogen interaction, Phagosome, and Phenylpropanoid biosynthesis. Compared to cd1 VS cd3, the gene ratios of these pathways are relatively higher in cd1 VS cd2, indicating that the pathways of Ribosome, Plant-pathogen interaction, Phagosome, and Phenylpropanoid biosynthesis are more easily induced and enriched in cd1 VS cd2. The unique enriched pathways in cd1 VS cd2 include Pyrimidine metabolism, Biosynthesis of various plant secondary metabolites, Phenylpropanoid biosynthesis, Purine metabolism, Arginine and proline metabolism, and Histidine metabolism. While in cd1 VS cd3, the specific enriched pathways are Photosynthesis - antenna proteins, Ascorbate and aldarate metabolism, Glyoxylate and dicarboxylate metabolism, Oxidative phosphorylation, Stilbenoid, diarylheptanoid and gingerol biosynthesis, Plant hormone signal transduction, Valine, leucine and isoleucine degradation, Flavonoid biosynthesis, Valine, leucine and isoleucine degradation, and Limonene and pinene degradation. Moreover, although stilbenoid, diarylheptanoid, and gingerol biosynthesis did not show significant enrichment in cd1 VS cd2, the DEGs encoding key enzymes in this pathway were significantly enriched and upregulated. Therefore, to further elucidate the antioxidant response mechanism of *G. bailinae* to Cd^2+^ stress, we chose to delve deeper into histidine metabolism and stilbenoid, diarylheptanoid, and gingerol biosynthesis, as some DEGs of them showed a clear and consistent trend of change.

### Pathways of L-histidine metabolism

**Figure 6:**
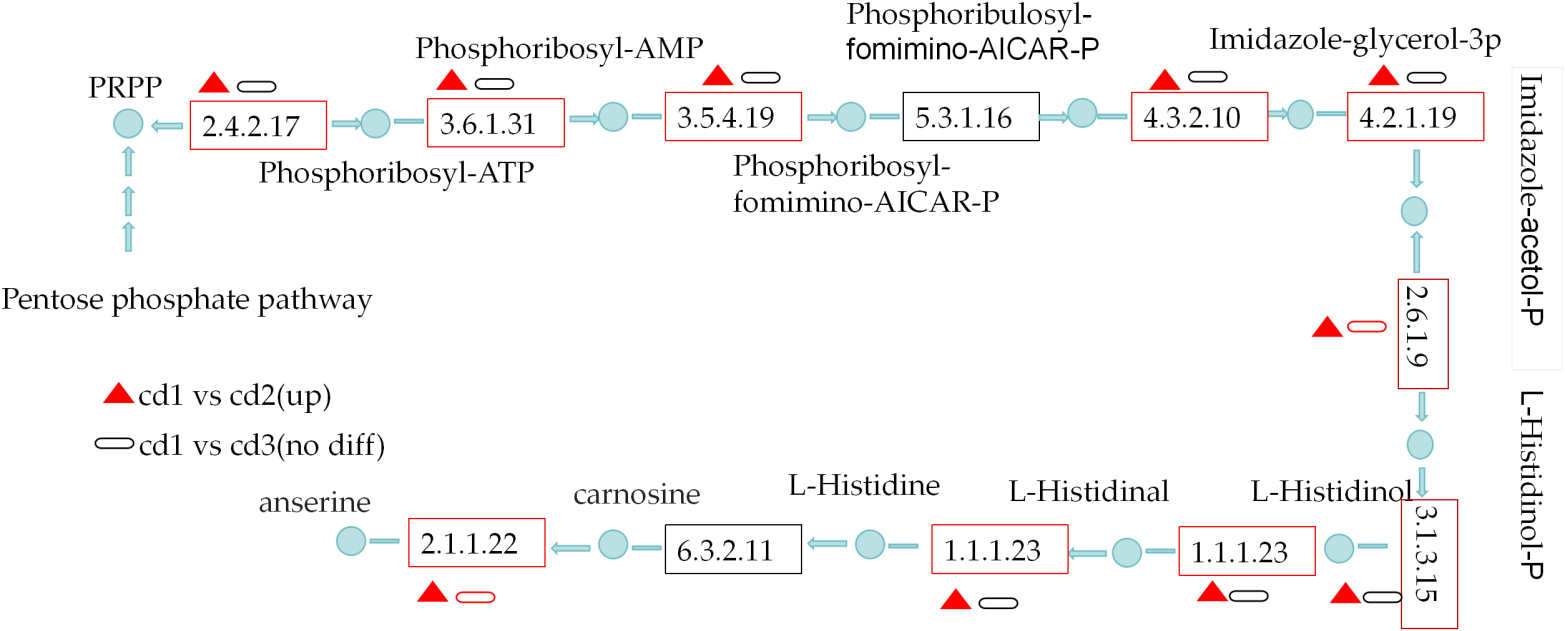
The L-histidine metabolism pathway analysis of *G. bailinae* in cd1 VS cd2 and in cd1 VS cd3.

Cd^2+^ significantly induced the pathways of L-histidine metabolism(ko00340) of *G. bailinae* in cd1 VS cd2. A total of eight key DEGs were enriched and upregulated in the L-histidine biosynthesis metabolism of *G. bailinae* in cd1 VS cd2, which encodes ATP phosphoribosyl transferase [EC:2.4.2.17], ATP phosphoribosyl transferase [EC:3.6.1.31], phosphoribosyl AMP cyclic hydrolase [EC:3.5.4.19], imidazole glycerol phosphate synthase [EC:4.3.2.10], imidazole glycerol phosphate dehydrase [EC:4.2.1.19], histamine phosphate transaminase [EC:2.6.1.9], histamine phosphatase [EC:3.1.3.15], histamine dehydrogenase [EC:1.1.1.23]. Moreover, The DEGs encodes carnosine n-methyltransferase [EC:2.1.1.22], histidine ammonia-lyase [EC:4.3.1.3] urocanate hydratase [EC:4.2.1.49] in L-histidine catabolic metabolism were all significantly upregulated (**Supplementary Table S4**) in cd1 VS cd2. None of the above DEGs of the L-histidine metabolism (ko00340) were highly enriched or up-regulated in cd1 VS cd3 (**Figure 6**).

### Pathways of stilbenoid, diarylheptanoid, and gingerol biosynthesis

**Figure 7:**
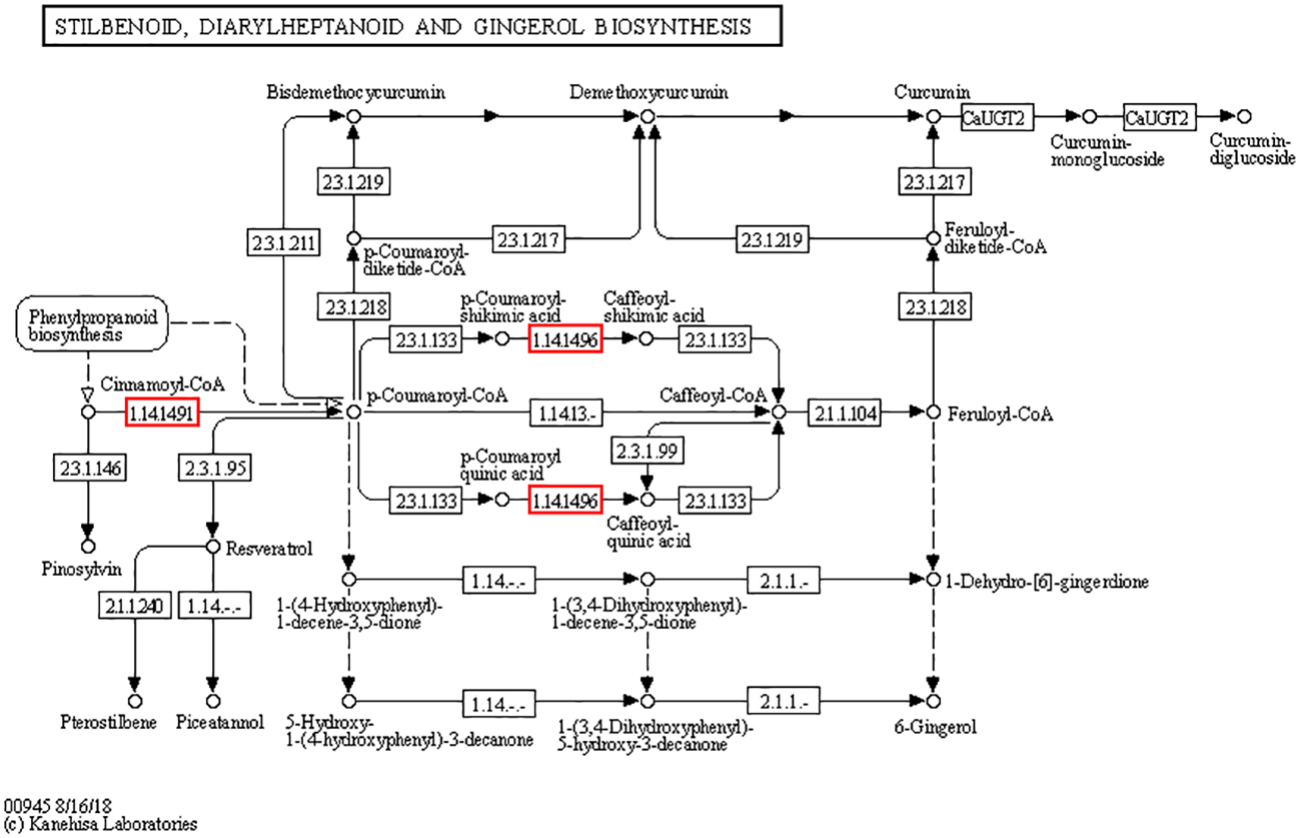
The stilbenoid, diarylheptanoid and gingerol biosynthesis pathways analysis of *G. bailinae.* The red boxes represent up-regulated expressed genes.

Cd^2+^ significantly induced the pathways of stilbenoid, diarylheptanoid, and gingerol biosynthesis (ko00945) both in cd1 VS cd2 and cd1 VS cd3. Three DEGs encodes transcinnamic acid 4-monooxygenase[EC:1.14.14.91], 5 -O-(4-coumaroyl)-D-quinate 3’-monooxygenase [EC:1.14.14.96] of stilbenoid, diarylheptanoid, and gingerol biosynthesis were significantly upregulated (**Figure 7**). Compared with cd1 VS cd3, The Unigenes of the above DEGs encoding transcinnamic acid 4-monooxygenase [EC:1.14.14.91] and 5-O-(4-coumaryl) -d-quinic acid 3’ -monooxygenase showed higher up-regulation in cd1 VS cd2 (**Table 1**). These results indicated that the biosynthesis pathways of stilbene, diarylheptane and ginger in *G. bailinae* were more active under low concentration (*P* ≤1 mg.L^-1^) of Cd^2+^ stress, while was limited under high concentration (*P*≥ 2.5 mg.L^-1^) of Cd^2+^ stress.

**Table 1:** The expression of DEGs in stilbenoid, diarylheptanoid and gingerol biosynthesis pathways of *G. bailinae*.

DEG	Symbol	Unigenes	Comparison group (log2FC)	
			cd1 VS cd2	cd1 VS cd3
1.14.1491	CYP37A	Unigene0016669	3.8	2.9
		Unigene0015754	5.5	4.6
		Unigene0005695	7.2	5.6
1.14.1496	CYP98A	Unigene0009237	5.1	4.1

## Discussion

The RGR of macroalgae is a direct reflection of their growth state (Jiang et al., 2009; Zhong et al., 2021). The soluble protein content in algae is an important index reflecting the level of plant metabolism (Xu et al., 2006). Most of these are functional proteins and enzymes involved in various metabolic processes, and certain soluble protein can also alleviate the toxic effect of heavy metals (Lü et al., 2017). Previous studies showed that the soluble protein content of algae can be increased by exposure to low Cd concentrations, while the synthesis and metabolism of proteins can be disrupted by high Cd concentrations (Tudoreanu and Phillips, 2004). Consequently, the soluble protein content of algae will be reduced. The results showed that *G. bailinae* in the cd2 treatment group maintained a stable RGR and had a higher soluble protein content than cd1 and cd3 treatment groups. The cd3 treatment group maintained a lower RGR and had less soluble protein contents than cd1 and cd2 treatments, indicating that *G. bailinae* has strong tolerance to low concentration of Cd^2+^ (≤1 mg·L^-1^). However, when the Cd^2+^ concentration exceeded a certain concentration threshold, a toxic effect and inhibition of the growth of algae would result. Research showed that RGR and soluble protein contents of *Gracilaria lemaneiformis*, *Hizikia fusiformis*, *Porphyra haitanensis*, and *Gracilaria lichenoides* (Zhu, 2010; Huang, 2013) showed the same trend with increasing exogenous Cd^2+^ concentration, which is consistent with the results of this study. At present, no unified conclusion has been reached regarding the reasons for the inhibition of algal growth by Cd exposure. The reason may be that Cd is an unnecessary element for algal growth, while algae can actively exclude or isolate Cd to reduce its toxicity, this consumes energy. The reduction of RGR caused by Cd stress may be related to the energy consumed for the adaptation and repair mechanisms of algae induced by Cd stress (Xia et al., 2004). Furthermore, substances formed by the combination of Cd and corresponding molecules in the algal body block certain basic functions of algae. Consequently, the absorption and transport of nutrient elements are inhibited, further leading to nutrient deficiency in algae (Kumar et al., 2010).

The MDA content reflects the damage degree of lipid peroxidation and membrane system caused by ROS (Weismann et al., 2011; Wang et al., 2014). Under normal conditions, the amount of antioxidant enzymes produced by the algal body is well controlled and maintains a state of dynamic equilibrium (Huang, 2013). However, when algae are stressed by heavy metals and other environmental factors, the activities of antioxidant enzymes such as SOD, CAT, and POD will increase to reduce the toxic effects of ROS accumulation (Wang et al., 2014). The results of this study show that during the same stress duration, the MDA content of *G. bailinae* increased with increasing Cd^2+^ concentration, while the activities of SOD, CAT, and POD first increased and then decreased. Significant differences were found (*P*< 0.05) among different Cd^2+^ concentration treatment groups, which is consistent with the results of *Hizikia fusiformis* and *Porphyra haitanensis* (Zhu, 2010). These results indicate that when the concentration of Cd^2+^ was low (≤ 1 mg·L^-1^), the toxic effect of Cd^2+^ on the algal body was dynamically reduced by increasing the activity of antioxidant enzymes such as SOD, CAT, POD and controlling the MDA content. However, if the Cd^2+^ concentration exceeds a certain threshold, the antioxidant enzyme activity of macroalgae can be inhibited (Dazy et al., 2009; Wu et al., 2009). This leads to the accumulation of MDA and ROS contents, and has a toxic effect on the algal body.

At present, transcriptomics of *G. bailinae* under stress have not been reported. Transcriptomic analysis data of *G. bailinae* are only available for different temperatures (Huang et al., 2023). Research showed that algae can resist or adapt to environmental changes by controlling their gene expression (Huang et al., 2023). Analysis of DEGs showed that more up-regulated genes were identified in cd1 VS cd2 than in cd1 VS cd3; Analysis of GO terms enrichment found that more GO entries involved in molecular function were enriched in cd1 VS cd2. which entries were mostly related to peptidase activity, endopeptidase activity, and ion transport. In the cd1 VS cd3 group, more GO items were enriched in biological processes, most of which were related to peptide biosynthetic and metabolism, as well as intracellular signal transduction. This result indicates that the related life activities of *G. bailinae* were more active under low Cd^2+^ concentration (≤1 mg·L^-1^) than under high Cd^2+^ concentration(≥ 2.5 mg·L^-1^). Low concentration of Cd^2+^ did not have substantial toxic effect on *G. bailinae*, the accumulation and discharge of Cd^2+^ in algae may be in a dynamic equilibrium state. High concentration of Cd produced substantial toxic effect on *G. bailinae*, and part of Cd^2+^ entered into the cell of algae and induced the synthesis and metabolism of peptides in the cell to reduce the toxic effect of Cd^2+^ (Hu et al., 2006). Moreover, KEGG enrichment of DEGs found that phenylpropane metabolism was significantly enriched both in cd1 VS cd2 and cd1 VS cd3 groups. Previous studies showed that this pathway could enhance the strength of the cell wall and plays an important role in plant resistance to environmental stressors, such as high salt levels, drought, and heavy metal exposure (Hu et al., 2013; Dong et al., 2015; Yadav et al., 2020). These results indicate that Cd^2+^ stress might activate the recognition and expression of phenylpropane-related genes of *G. bailinae*, thereby enhancing the adsorption and accumulation capacity of Cd^2+^ in the algal cell wall, and further preventing Cd^2+^ from entering algal cells.

Histidine is a precursor substrate for the synthesis of alpha-ketoglutaric acid, which can eliminate oxidative stress through non-enzymatic reactions (Lemire et al., 2010; Song et al., 2022). Its dipeptide compounds such as carnosine and anserine are important antioxidant substances that can inhibit the production of ROS by scavenging free radicals (Wang et al., 2022). Song et al. (2022) found that histidine biosynthesis plays an important role in Mn(II) tolerance by increasing intracellular alpha-ketoglutarate levels of *Stenotrophomonas* sp. It has also been shown that histidine improved the tolerance of *Caenorhabditis elegans* and *Aspergillus fumigatus* to heavy metals (Murphy et al., 2011; Dietl et al., 2016; Song et al., 2022). These mechanisms have recently also been identified in plants. Ji (2023) confirmed that histidine supplementation can improve the activities of antioxidant enzymes such as SOD, POD, and CAT as well as the contents of the soluble protein in corn roots under environmental stress. In this study, the expression of genes related to histidine biosynthesis was significantly upregulated, especially in the cd1 VS cd2 group. Up-regulated genes included eight DEGs encoding the key enzymes of L-histidine biosynthesis and DEGs encoding the key enzymes of carnosine methylation. It can be speculated that under low Cd^2+^ concentration, *G. bailinae* might increase the level of intracellular α-ketoglutaric acid through accumulating histidine; then, the activities of SOD, POD, and CAT as well as the content of anserine and soluble protein are increased to reduce the accumulation of ROS and alleviate the damage caused by Cd^2+^ stress.

Previous studies have shown that stilbenes, diarylheptane, and gingerol have various biological efficacies, including antioxidant and antiallergic activities (Semwal et al., 2015). Ouyang et al. (2023) found that the down-regulation of these pathways in chili roots under Cd exposure correlated with a decrease of antioxidant and antibacterial activities in chili roots. The results of this study showed that three DEGs encodes transcinnamic acid 4-monooxygenase[EC:1.14.14.91], 5 -O-(4-coumaroyl)-D-quinate 3’-monooxygenase [EC:1.14.14.96] were significantly upregulated. Compared with cd1 VS cd3, The Unigenes of the above DEGs encoding transcinnamic acid 4-monooxygenase [EC:1.14.14.91] and 5-O-(4-coumaryl) -d-quinic acid 3’ -monooxygenase showed higher up-regulation in cd1 VS cd2. In the phenylpropanoid pathway of plants, p-Coumaroyl-CoA is catalyzed to form lignin by direct reduction of cinnamoyl-CoA reductase and cinnamyl alcohol dehydrogenase. Caffeoyl shiknic acid can also be catalyzed to produce caffeic acid by caffeoyl shikimate esterase, which then enters the synthetic lignin synthesis pathway (Vanholme et al., 2012). Lignin—one of the main components of the secondary plant cell wall—plays an important role in the fixation of heavy metal ions. It can reduce the migration ability of heavy metal ions in plants, inhibit the entry of heavy metal ions into the cytoplasm, and enhance the tolerance of plants to heavy metal stress (Ge et al., 2023). Caffeoyl quinic acid plays an important role in plant resistance physiology, as it can improve the ability of plants to eliminate ROS and free radicals and defend against both biological and abiotic stresses (Pandey et al., 2014). Therefore, in the present study, Cd^2+^ stress induced an upregulation of DEGs of the stilbenoid, diarylheptanoid, and gingerol biosynthesis pathway to increase the expressions of p-Coumaroyl-CoA, caffeoyl quinic acid, and caffeoyl shiknic acid. Consequently, the lignin synthesis and antioxidant capacity of *G. bailinae* under Cd treatment were improved. Although the stilbenoid, diarylheptanoid, and gingerol biosynthesis was enriched in both cd1 VS cd2 and cd1 VS cd3 groups, the expressions of the three DEGs encoding 1.14.1491 (CYP73A) and 1.14.1496 (CYP98A,C3’H) were more upregulated in cd1 VS cd2 than in cd1 VS cd3 groups. These results indicate that *G. bailinae* could not only maintain a stable growth state by upregulating the key DEGs of the stilbenoid, diarylheptanoid, and gingerol biosynthesis pathway under low concentration of Cd^2+^ (≤1 mg·L^-1^) stress, but also maintain a basic life state under high Cd^2+^ concentration (≥2.5 mg·L^-1^) stress.

Cd phytotoxicity is commonly associated with the extent of its accumulation in plants (White and Brown, 2010). Considering the duration of stress exposure, the results of this study also show that the RGR, soluble protein and MDA contents, SOD, CAT, and POD activities of *G. bailinae* remained relatively stable in cd2 treatment group with extended stress duration. No significant differences (*P* > 0.05) were found among different Cd^2+^ stress duration, and the toxic effect of Cd^2+^ on the algal body was dynamically reduced by increasing the activity of antioxidant enzymes such as SOD, CAT, POD and controlling the MDA content, Even the accumulation and excretion of Cd^2+^ in the algae may have reached a certain dynamic equilibrium. However, the MDA content of the cd3 treatment group increased with extended stress duration, while the RGR, soluble protein contents, and the activities of SOD, CAT, and POD decreased, and significant differences (*P*< 0.05) were found among different stress duration. These results might indicate that *G. bailinae* was able to maintain a stable growth state in the low concentration group (≤1 mg·L^-1^), while continued to deteriorate in the high concentration group (≥2.5 mg·L^-1^). And these results further supports that *G. bailinae* can withstand low concentration (≤1 mg·L^-1^) Cd^2+^ stress and is not affected by the duration of the stress, and the toxicity of high Cd^2+^ concentration on *G. bailinae* has a time cumulative effect, the degree of which may be negatively correlated with the concentration and stress duration of Cd^2+^. Consequently, the algal body was able to maintain a stable growth state. However, if the Cd^2+^ concentration exceeds a certain threshold, the antioxidant enzyme activity of macroalgae might be inhibited (Dazy et al., 2009; Wu et al., 2009), which leads to the accumulation of MDA and ROS contents, and has a toxic effect on the algal body.

We inferred that under low-concentration cadmium ion (≤1 mg·L^-1^) stress, *G. bailinae* possesses a stable tissue tolerance mechanism to cope with Cd^2+^ stress duration. This mechanism is facilitated by upregulating DEGs and enriching GO terms related to peptidase activity, endopeptidase activity, and ion transport, enabling the algae to maintain a dynamic balance between the accumulation and excretion of Cd^2+^. By enriching and upregulating the histidine metabolic pathway, the histidine content of the algae is increased, leading to enhanced activities of SOD, POD, and CAT, as well as increased the levels of anserine and soluble protein. This helps effectively eliminate ROS and keep the MDA content at a relatively low and stable level. Enriching and upregulating DEGs in the phenylpropanoid pathway, stilbenoid, diarylheptanoid, and gingerol biosynthesis pathways promotes lignin synthesis and the regeneration of the algal cell wall, enhancing the adsorption and accumulation capacity of the algal cell wall for Cd^2+^ and further preventing Cd^2+^ from entering the algal cells. However, under high-concentration (≥2.5 mg·L^-1^) Cd^2+^ stress, this stable tissue tolerance mechanism is disrupted. The accumulation of Cd^2+^ in the algae exceeds its excretion, allowing some Cd^2+^ to enter the algal cells, inhibiting the growth state of the algae. By upregulating DEGs and enriching GO terms related to peptide biosynthesis and metabolism, as well as the intracellular signal transduction, along with DEGs in the phenylpropanoid pathway, stilbenoid, diarylheptanoid, and gingerol biosynthesis pathways, the algae enhance their ability to detoxify cellular toxicity, promote lignin and cell wall regeneration, enabling *G. bailinae* to maintain basic life activities.

## Conclusion

This study revealed that the RGR and soluble protein contents of *G. bailinae* remained unaffected under low Cd^2+^ concentration stress (≤1 mg·L-1), but were significantly inhibited under high Cd^2+^ concentration stress (≥2.5 mg·L-1). Under low Cd^2+^ concentration, there was no substantial toxic effect on the algal body due to the ability of *G. bailinae* to dynamically eliminate the toxic effects of Cd^2+^ by enhancing the activities of antioxidant enzymes such as SOD, CAT, and POD, and controlling the MDA content. However, under high Cd^2+^ concentration, the activities of these antioxidant enzymes were inhibited, leading to increased MDA content and a substantial toxic effect on the algal body. The transcriptome analysis showed that under low Cd concentration stress (≤1 mg·L^-1^), more GO terms related to peptidase activity, endopeptidase activity, and ion transport were enriched, potentially facilitating a balanced state of Cd^2+^ accumulation and excretion. Under high Cd concentration stress (≥2.5 mg·L^-1^), more GO terms related to peptide biosynthesis and metabolism, as well as intracellular signal transduction, were enriched. This suggests that as Cd^2+^ entered the algal cells, it induced the synthesis and metabolism of peptides to mitigate its toxic effects. Furthermore, the phenylpropane metabolism pathway was significantly enriched, which helps enhance cell wall strength and improve the algal body’s tolerance to Cd^2+^ stress. Additionally, *G. bailinae* modified the L-histidine metabolism pathway and the stilbenoid, diarylheptanoid, and gingerol biosynthesis pathways, which are involved in antioxidant synthesis and lignin synthesis. This modification enhanced antioxidant enzyme activity and cell wall strength, preventing Cd^2+^ from entering the algal cells and reducing MDA accumulation. These findings provide a deeper understanding of the antioxidant response mechanism of *G. bailinae* to different concentration and stress duration of Cd^2+^, which can serve as a basis for future academic research in this field. However, further in-depth research is still needed to understand how the enrichment of histidine metabolic pathways in the algae promote the enhancement of antioxidant enzymes, which will be the focus of future related studies.

## Data availability statement

The datasets presented in this study can be found in online repositories. The names of the repository/repositories and accession number(s) can be found below: BioProject, PRJNA1071728.

## Author contributions

ZL: Writing – original draft, Writing – review & editing. YL: Methodology, Writing – original draft. EX: Investigation, Writing – original draft. YS: Writing – review & editing.
