# Supplementary material for: Transcriptome analysis discloses antioxidant detoxification mechanism of Gracilaria bailinae under different cadmium concentrations and stress durations
Source: Front Plant Sci. 2024 Jul 5;15:1371818. doi: 10.3389/fpls.2024.1371818 (PMC11257999; doi:10.3389/fpls.2024.1371818)
Supplement: Supplementary file 1 [file DataSheet_1.pdf]

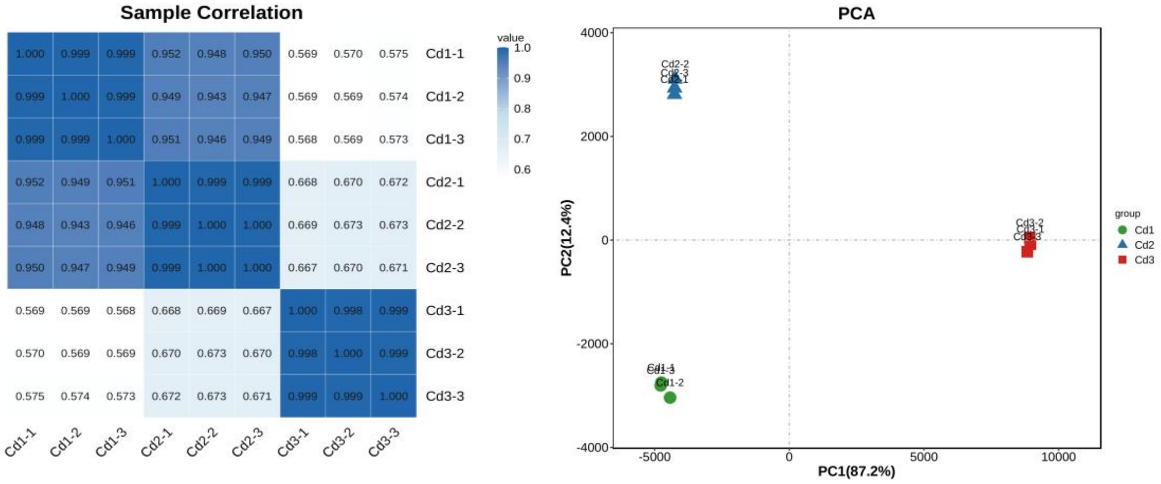

A B

FIGURES1

Sample correlation analysis. (A) Heat map of Pearson correlation coefficients. (B) Two-dimensional diagram of principal component analysis

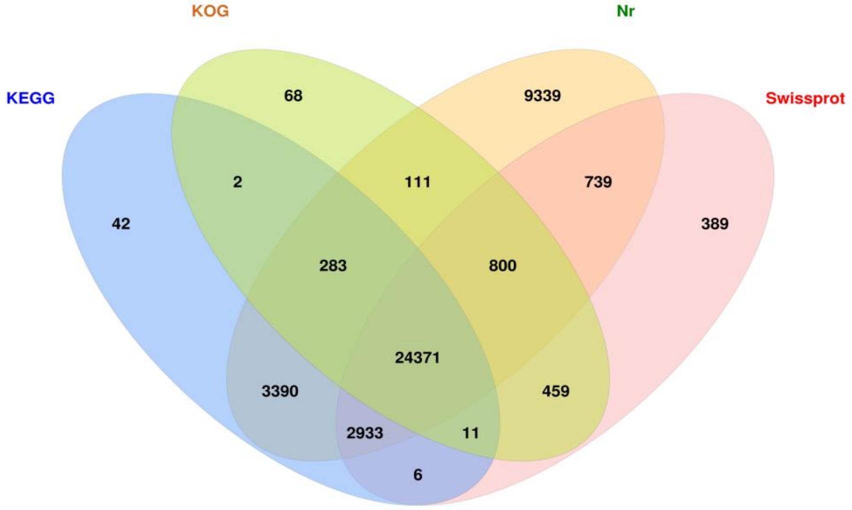

FIGURE S2

Four major databases annotated with Venn diagrams for the transcriptome of *G. bailinae*

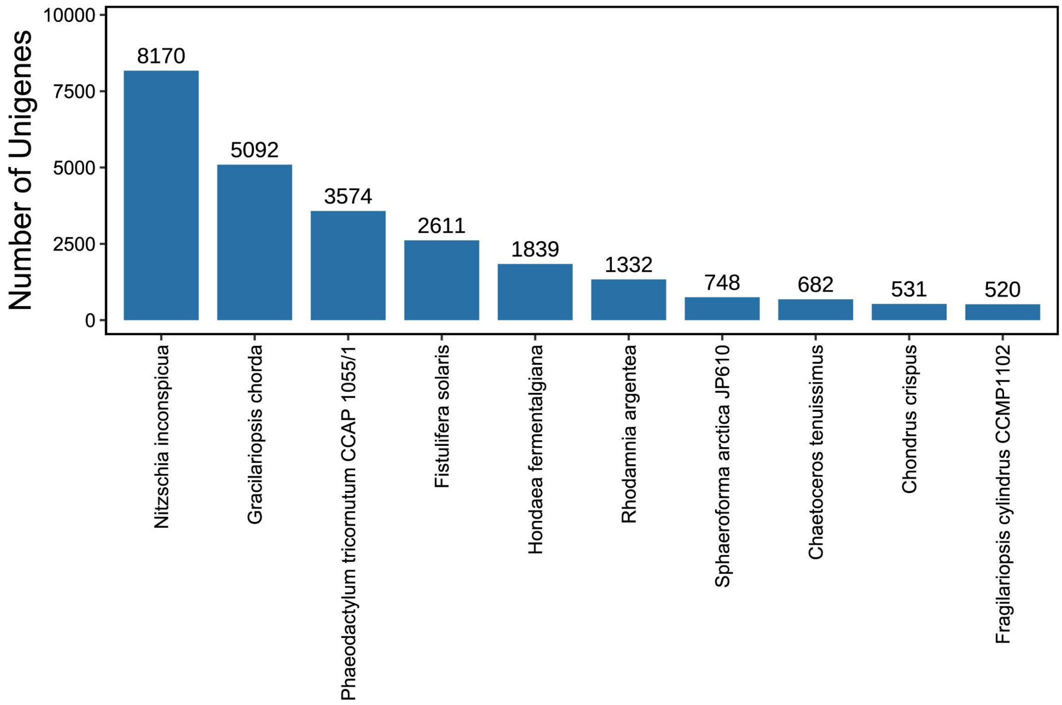

FIGURE S3

Statistics of the number of species compared from the Nr database based on the transcriptome results of *G. bailinae*.

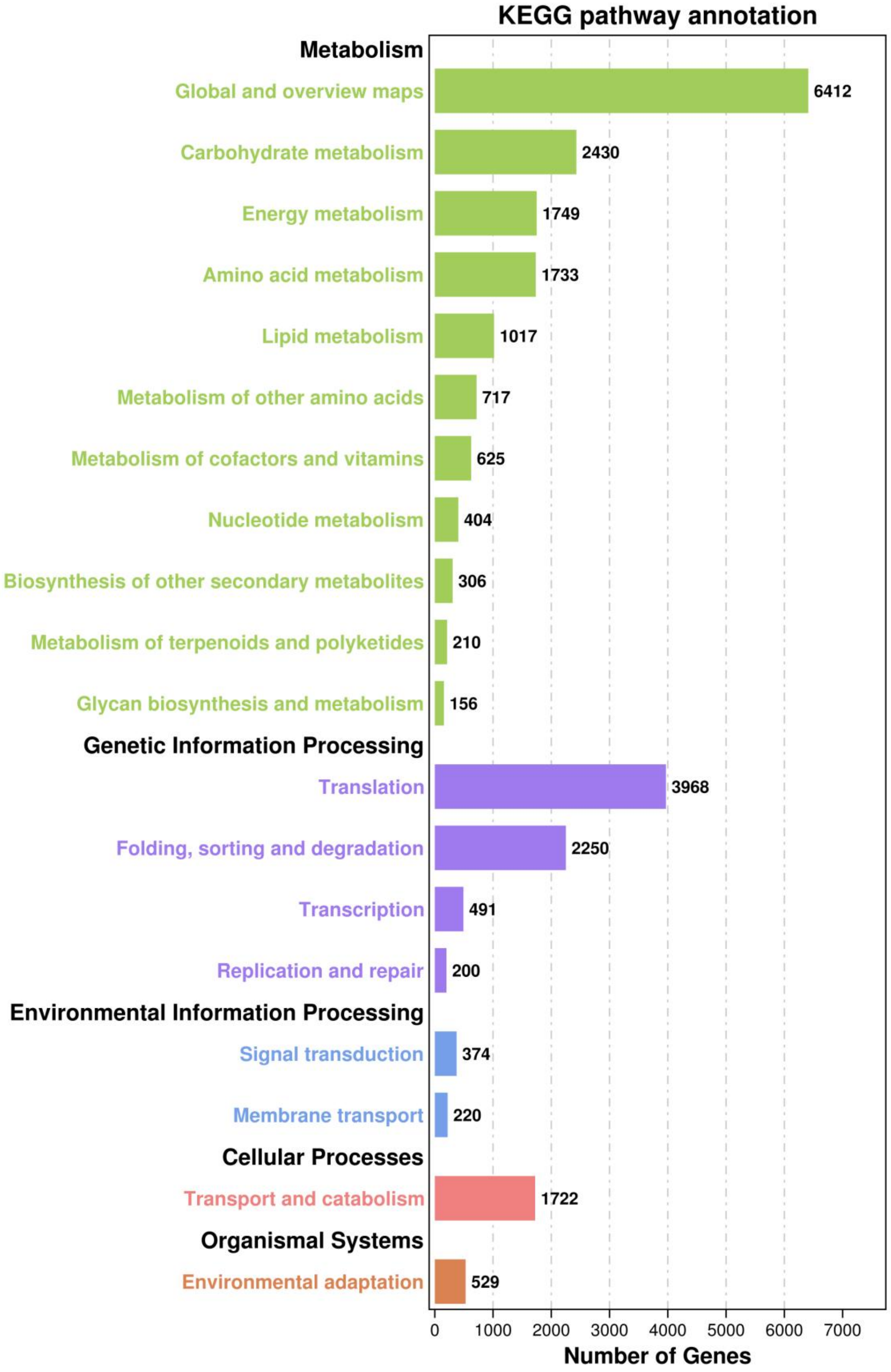

FIGURE S4

Statistics of Pathway results annotated from the KEGG database based on the transcriptome results of *G. bailinae*.

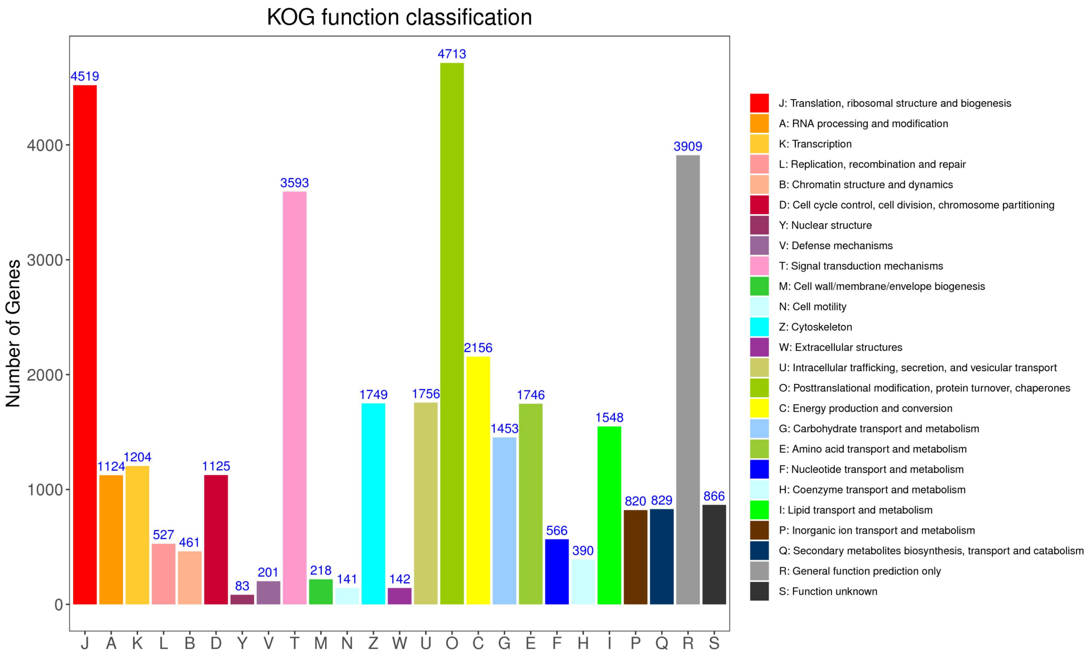

FIGURE S5

Statistics of KOG functional classification results annotated from the COG/KOG database based on the transcriptome results of *G. bailinae*.

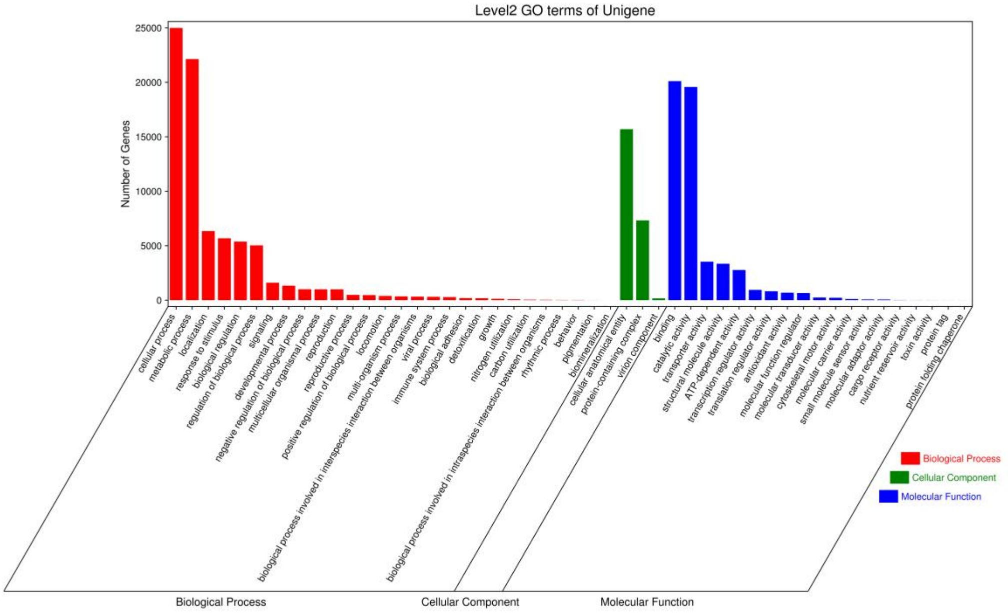

FIGURE S6

Gene Ontology (GO) Level 2 Classification Statistics for the transcriptome of *G. bailinae*.
